# End-Grafted Polymer Chains onto Inorganic Nano-Objects

**DOI:** 10.3390/ma3031981

**Published:** 2010-03-18

**Authors:** Demetra S. Achilleos, Maria Vamvakaki

**Affiliations:** 1 Institute of Electronic Structure and Laser, Foundation for Research and Technology–Hellas, P. O. Box 1527, 711 10 Heraklion, Crete, Greece; E-Mail: achill@iesl.forth.gr (D.S.A.); 2 Department of Materials Science and Technology, University of Crete, P. O. Box 2208, 710 03 Heraklion, Crete, Greece

**Keywords:** nanohybrids, core-shell nanoparticles, end-grafted polymers

## Abstract

Organic/inorganic nanohybrid materials have attracted particular scientific and technological interest because they combine the properties of the organic and the inorganic component. Inorganic nanoparticles exhibit interesting electrical, optical, magnetic and/or catalytic properties, which are related with their nano-scale dimensions. However, their high surface-to-volume ratio often induces agglomeration and leads to the loss of their attractive properties. Surface modification of the inorganic nano-objects with physically or chemically end-tethered polymer chains has been employed to overcome this problem. Covalent tethered polymer chains are realized by three different approaches: the “grafting to”, the “grafting from” and the “grafting through” method. This article reviews the synthesis of end-grafted polymer chains onto inorganic nanoparticles using “controlled/living” polymerization techniques, which allow control over the polymer characteristics and the grafting density of the end-tethered polymer chains.

## 1. Introduction

The synthesis of core-shell nano-objects comprising of an inorganic core and a polymer shell has recently gathered great scientific interest with a view towards new opportunities for constructing functional nanostructured materials [1,2]. Inorganic-polymer core-shell structures combine the fascinating electronic, optical and magnetic properties of the core with the desired properties of the polymer such as solubility, film formation, processability and compatibility to the environment [3,4]. Several types of nanoparticles have been employed such as silica, metal, magnetic and semiconducting colloids, which depending on their composition and ordering, can find size-depended fascinating applications in a variety of technologies such as diffractive optics, electro-optical devices, information storage and reinforced composite materials. On the other hand, advances in polymer chemistry have allowed control over the chemical composition and function of the polymer shell that governs the interactions with the surrounding media, leading to complex self-organization phenomena in core-shell systems. 

The intense interest towards the modification of the inorganic nanomaterials surface with polymer chains has been fueled by the continual tendency of the former to agglomerate due to the strong adhesion forces among the particles originated from their high surface-to-volume ratio. Most of the properties of the nanoparticles are dictated by their nano-sized dimensions and are dramatically altered upon aggregation. Polymer-coated particles are sterically stabilized in interparticle lengths scales in the nanometer range compared to the shorter separation distances accessed by short alkyl chain stabilizers. Moreover, the polymer coating has a lot of free volume, which not only improves the dispersion stability of the nanoparticles in organic and aqueous media, but can also prevent the segregation of the inorganic fillers in polymer matrices due to the high affinity of the particle modifier for the organic medium. Two different approaches have been employed for the preparation of polymer-coated nanoparticles; the first involves the reversible physisorption of the polymer chains onto the nanoparticles surface *via* weak interactions (van den Waals forces, hydrogen bonds, *etc.*) and the second the permanent attachment of the macromolecular chains onto the nanoparticles surface *via* chemisorption. The covalent binding of the polymeric modifiers onto the curved surface can be achieved either by the “grafting to” method, upon which pre-prepared macromolecules are grafted onto the nanomaterials surface using the affinity of the polymer end-group for the functionalities present on the surface of the particles [5], or by the *in situ* growth of the polymer chains from appropriately functionalized nano-objects known as the “grafting from” method. 

Several polymerization techniques such as living anionic [3], living cationic [6,7,8,9,10], ring-opening polymerization (ROP) [11,12,13,14,15], ring-opening metathesis polymerization (ROMP) [16,17] and controlled/“living” radical polymerization (CRP) have been employed for the irreversible functionalization of inorganic nano-materials with end-tethered polymer chains. Among them, the development of the CRP techniques has offered lately great potential for the synthesis of well-defined core-shell nanostructures. CRP techniques, namely atom-transfer radical polymerization (ATRP), reversible addition-fragmentation chain transfer polymerization (RAFT) and nitroxide-mediated radical polymerization (NMP), have attracted considerable attention because they provide a facile approach for the development of end-grafted macromolecules and a high degree of synthetic flexibility towards the introduction of a variety of functional polymers onto the surface of different nanoparticles. CPR techniques also allow the covalent attachment of macromolecules with well defined grafting densities and controlled film thicknesses. Densely packed arrays of polymer chains can be attained which are forced to adopt a stretched conformation, due to excluded volume interactions, leading to coatings of high thicknesses [18].

However, there are certain advantages and limitations inherent to each of the above methods. In ATRP the initiators, transition metal catalyst systems and ligands used are commercially available. An important advantage of ATRP is the polymerization of a wide range of monomers including not only the more reactive meth(acrylates), styrenes and acrylonitrile, but also less reactive monomers such as vinyl acetate and vinyl chloride [19]. However, a problem often encountered in ATRP is the complete removal of the transition metal catalyst from the final product which can be critical for certain applications, *i.e.*, in electronics. In contrast, NMP and RAFT do not employ metal catalyst systems however, the commercial availability of alkoxyamines and dithioesters used as radical capping agents is quite limited. Moreover, RAFT is incompatible with basic monomers, while the NMP of methacrylates and other less reactive molecules is still challenging [20].

In this review, the focus is on the surface modification of inorganic nanoparticles using the “grafting to” and “grafting from” techniques to prepare uniform core-shell nanostructures. The synthesis, properties and potential applications of the hybrid structures are reviewed for six different types of nanoparticles including, silica, silsequioxane, metal-oxide semiconducting, magnetic, metal and quantum dots. Particular emphasis is given on responsive polymer coatings which endow the inorganic nanoparticles with new exceptional physical and chemical properties.

## 2. Results and Discussion

### 2.1. Polymer-grafted silica nanoparticles

#### 2.1.1. “Grafting to” method

**Figure 1:**
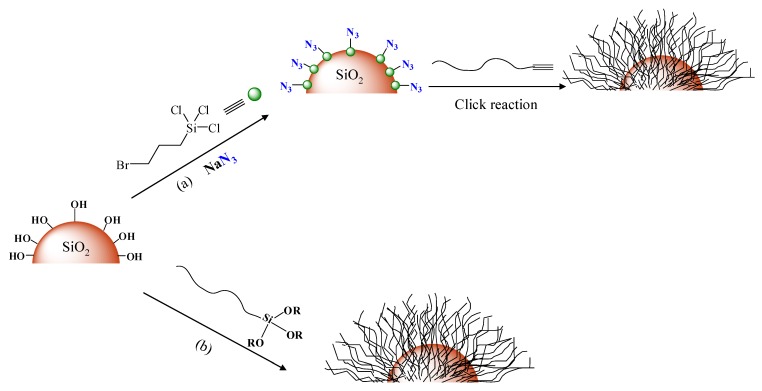
Schematic representation of the synthetic procedure followed for the grafting of polymer chains onto the surface of silica particles *via* the “grafting to” approach using click chemistry (a) and silane coupling (b).

Silica particles are widely used as reinforcing fillers to improve the mechanical and thermal properties of a polymer matrix. They can be easily prepared by the Stöber method [21], which allows control over the nanoparticle size and size distribution. The grafting of polymer chains onto the surface of silica nanoparticles has been achieved by the synthesis of linear macromolecules using a controlled/living polymerization technique followed by organic reactions such as Huisgen cycloaddition and other coupling reactions to immobilize the polymers onto the nanoparticle surface (Figure 1). Click chemistry has been used successfully employed to immobilize alkyne-terminated homopolymers and diblock copolymers synthesized by RAFT and/or ATRP onto the surface of azide-modified silica particles [22]. The same strategy was successfully applied to load mesoporous silica with polymers without blocking the pores of the inorganic material [23]. A more direct method which does not require the pre-functionalization of the inorganic nanoparticles employed a combination of RAFT polymerization and siloxane coupling reactions to functionalize the surface of silica nanoparticles with pre-synthesized polymer chains. In this work, trimethoxysilane-functionalized homopolymers and block copolymers were first synthesized by RAFT polymerization mediated by S-methoxycarbonylphenylmethyl S’-trimethoxysilylpropyltrithiocarbonate and were linked onto the surface of the nanoparticles *via* the coupling reaction of the hydroxyl groups of the solid surface with the trimethoxysilane end-groups of the macromolecules [24]. 

An interesting approach used the covalent layer-by-layer (LbL) assembly of acetylene- and azido-functionalized poly(N-isopropylacrylamide) PNIPAM random copolymers synthesized by ATRP onto silica nanoparticles to prepare ultrathin thermo-responsive microcapsules *via* click chemistry. PNIPAM is a thermo-responsive polymer, which responds to changes of the solution temperature and exhibit the so-called lower critical solution temperature (LCST). The LCST corresponds to the region in the phase diagram of a polymer at which the enthalpic contribution of hydrogen-bonded water to the polymer chain becomes lower than the entropic gain of the system as a whole. At this point the polymer chains undergo a coil-to-globule transition due to hydrophobic interactions and phase separate [25,26]. The LbL assembly was based on the 1,3-dipolar cycloaddition of the azide groups located at the surface of the silica particles with the acetylene-functionalities of a PNIPAM copolymer in the solution. The remaining free acetylene groups located at the outermost surface polymer layer were reacted with an azido-functionalized PNIPAM random copolymer in the next assembly step leading to the LbL formation. Upon etching the silica core of the assembly with HF hollow PNIPAM microcapsules that exhibited reversible thermo-induced swelling/deswelling behavior were obtained, which are particularly attractive for use on controlled drug delivery applications [27].

#### 2.1.2. “Grafting from” approach

##### 2.1.2.1. “Hairy” homopolymer and block copolymer silica particles

**Figure 2:**
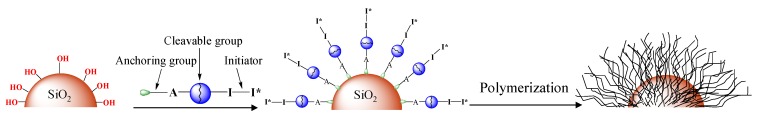
Schematic representation of the synthetic procedure followed for the preparation of silica-polymer core-shell hybrids using the “grafting from” method.

The surface hydroxyl groups exposed on the periphery of silica permit their facile derivatization to appropriate initiating moieties (Table 1) for the controlled growth of polymer chains from the surface of the nanoparticles (Figure 2). The si-ATRP from silica nanoparticles was first reported by Patten *et al.* to polymerize polystyrene (PS) and poly(methyl methacrylate) PMMA end-grafted chains [28,29]. Since then, a variety of polymeric stabilizers including homopolymers and copolymers of styrenes, meth(acrylates) and acrylamides have been grown from the surface of spherical silica colloids [30,31,32,33,34,35,36,37,38,39]. Recently, the controlled polymerization of dense PMMA chains, with a very narrow molecular weight distribution from a silica core was achieved by ATRP in bulk. A high grafting-density hydrophobic brush was successfully prepared using an initiator carrying a triethoxysilane anchor group [40]. The control of the grafting density resulted in the formation of a colloidal crystal when the hybrids were suspended in a good solvent for the polymer in a certain concentration range which decreased with increasing the graft polymer chain length, *L*_c_. Long polymer chains led to a nearest neighbor interparticle distance *D*_dis_ within the crystal in the micrometer length scale. The colloidal crystal included both hexagonal close-packed (hcp) and face-centered cubic (fcc) lattice arrangements with the fcc arrangement likely to increase when increasing the polymer chain length. This transition of the crystalline structure from a nearly random stacking to an fcc arrangement was ascribed to a qualitative change of the conformation of the grafted chains and hence the interparticle potential curve at the crossover of the concentrated polymer brush to the semidilute polymer brush in the effective graft density of the polymer layer [41,42]. Moreover, the grafting density of the polymer had a significant effect on the “softness” of the hybrid colloids. Significant interpenetration of the grafted polymer chains took place for particles in the intermediate brush regime and decreased as the polymer grafting density increased. As a result, the threshold concentration for graft polymer interpenetration of dense particle brushes was found to increase by about an order of magnitude as compared to the intermediate brush analogue [43,44]. These finding offer unique possibilities for fundamental and applied research of colloidal systems for application in photonic materials, sensors and elsewhere. 

**Table 1:** Initiator molecules grafted on the surface of silica particles.

No	Initiator	Ref.
1	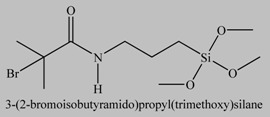	[49,53,54,55,56,57,58,59,60]
2	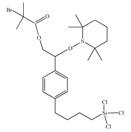	[61,62]
3	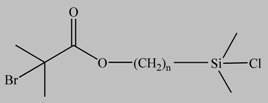	[63,64,65,66]
4	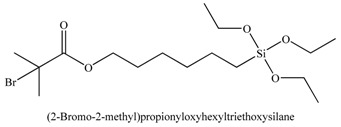	[40,67]
5	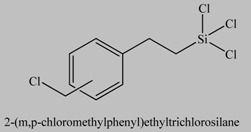	[68,69,70,71]
6	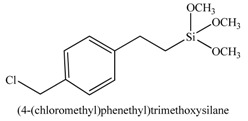	[72]
7	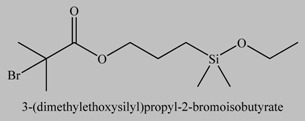	[45,46]

The synthesis of hydrophilic methacrylate polymers from the surface of silica particles *via* ambient temperature ATRP in protic media, utilizing a siloxane-grafted initiator [45], have been shown to suffer from the hydrolytic cleavage of the grafted polymer chains from the nanoparticle surface [46]. To overcome this problem and circumvent the need for surface pre-treatment of the silica sol with siloxane initiators prior to the polymerization, a cationic ATRP initiator was electrostatically absorbed onto ultrafine anionic silica for subsequent polymer grafting of hydrophilic methacrylates in protic media at ambient temperature. Nonionic and cationic monomers have been polymerized directly in one-pot syntheses with reasonably high conversions, however the growth of anionic polymers was hindered due to the electrostatic interactions between the negatively charged monomer with the cationic macroinitiator-particles [47,48]. Furthermore, this approach also allowed the synthesis of zwitterionic polymer coatings [49] which are highly resistant to nonspecific protein adsorption in complex media and improve the bio-interfacial properties of the silica particles protecting them against aggregation in complex physiological conditions [50]. 

Another hybrid system was developed by the si-ATRP of acrylonitrile from the surface of silica nanopartricles. The “hairy” silica-polyacrylonitrile (PAN) hybrids were used as precursors for the synthesis of nanoporous carbon thin films with appreciable adsorption capacities after the pyrolysis of the PAN stabilizers, followed by the removal of silica by HF etching [51]. Using a similar protocol, mesoporous carbons were obtained by the surface polymerization of acrylonitrile from ordered and disordered mesoporous silica templates carrying surface initiating sites followed by the stabilization of the polymer layer under air and its subsequent carbonization at higher temperatures [52]. 

Silica-polymer core-shell hybrids are particularly advantageous for the facile preparation of uniform hollow polymer microspheres by the removal of the silica core of the hybrids using HF etching. Homopolymer and block copolymer coated particles have been used as precursors to obtain polymeric hollow spheres of uniform size and good dispersibility in aqueous or organic media. The structural integrity of the hollow shell or capsule is preserved by either cross-linking the polymer chains [67,73], or by dispersing the hollow structure in a bad solvent for the polymer [72]. Such hollow capsules are very attractive for the encapsulation and controlled delivery of active compounds in drug delivery and other biomedical applications.

NMP is based on a similar concept to that of ATRP involving the activation–deactivation equilibrium between dormant species and a small fraction of propagating macroradicals. Although this technique has not been extensively employed as a surface-initiated method there are a few examples of alkoxyamine systems in the literature used to polymerize homopolymers and diblock copolymers of styrenes and meth(acrylates) from the surface of silica nanoparticles [74,75,76,77,78,79,80,81,82,83,84]. In order to overcome the complicated synthesis of specific surface grafted NMP initiators an alternative method, that of “trapping of carbon radicals” has been used [85,86] which provides the feasible trapping of the growing carbon radicals by the NMP initiator. However, this method suffers from secondary reactions, such as cross-linking or formation of inactive molecules, which reduce the efficiency of the grafting procedure. The initiating process by the *in situ* trapping of the carbon radicals is also hindered by the presence of unreacted double bonds on the surface of the nanoparticles upon grafting the initiator. Thus, an alternative approach, the “*in situ* thermo-dependant trapping of the carbon radicals,” has been developed. This method provides good control over the molecular weight and the grafting density of the bound macromolecules leading to hybrid suspensions with long-term colloidal stability [76,87].

Well-defined hybrid silica-polymer core-shell structures have been synthesized as well *via* the RAFT technique. The application of this versatile method to prepare polymer brushes on the surface of silica nanoparticles is unproportionally limited compared with the large number of studies on the surface initiated-ATRP (si-ATRP) from silica particles. Although the RAFT technique is compatible with the polymerization of almost all the conventional monomers, the covalent attachment of the RAFT agents onto the nanoparticles surface is still challenging thus restricting its utilization. Most studies in the literature investigate the anchoring of the RAFT agents onto the nanoparticles surface. Silane chemistry [88,89] and the condensation of mercaptothiazoline-activated RAFT agents with amino groups present of the nanoparticles surface [90] have been the most successful methods at present. For the RAFT polymerization of styrenics and (meth)acrylates on solid supports two different approaches have been utilized; the R-group approach [88,89,90,91,92] and the Z-group approach [93,94]. In the R-group approach the chain transfer agent (Table 2) is attached to the backbone *via* the leaving and reinitiating R-group, while in the Z-group approach the chain transfer agent is attached to the backbone *via* the stabilizing Z-group. Both methods exhibit advantages and limitations. For instance, the R-group approach can provide grafted polymers of higher molecular weight and grafting density however, the molecular weight distribution of the grafts may be broadened by possible side reactions such as chain coupling. On the other hand, the Z-group approach can yield well-defined grafted polymers with monomodal molecular weight distributions but of lower grafting densities due to shielding effects.

**Table 2:** RAFT agents bound on silica nanoparticles (R-group approach).

No	RAFT agent	Polymer	Ref.
1	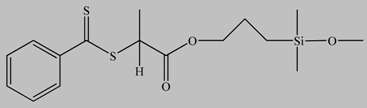	P*n*BA, PS-b-P*n*BA	[88]
2	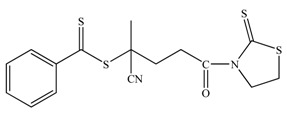	PMMA	[90]
3	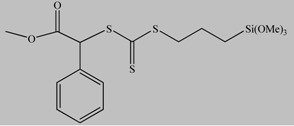	PMA, P*n*BA, PMMA, PS, PNIPAM, PDMA	[89]
4	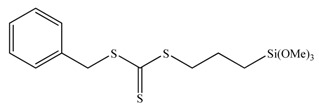	PMA, P*n*BA, PMMA, PS, PNIPAM, PDMA	[89]

Finally, living-anionic surface-initiated polymerization has been also successfully employed for the growth of PS chains from the surface of silica nanoparticles pre-functionalized with a 1,1-diphenylethylene derivative bearing chlorosilane end-groups. Although a living anionic polymerization mechanism was reported, attributed to the linear increase of the molecular weight of the bound polymer upon increasing the amount of monomer, the excess initiator present in solution and the observed nanoparticle aggregation limit the applicability of this method for the preparation well-defined core-shell structures [95]. 

##### 2.1.2.2. Grafted comb and branched polymer architectures

Polymer brushes of more complex architectures can be grown effectively from the surface of silica nanoparticles. One approach for the synthesis of comb-coil polymer brushes from the nanoparticle surface employed a three step reaction; first poly(2-hydroxyethyl methacrylate) (PHEMA) brushes were grown from the surface of initiator-functionalized silica particles by ATRP, followed by the ring-opening polymerization of DL-lactide initiated by the hydroxyl side-groups of PHEMA in the second step and the activators generated by electron transfer (AGET) ATRP of an acrylic monomer initiated by the bromine end-groups of the PHEMA chains in the final step [55]. Alternatively, a more convenient method involving a one-step reaction for the preparation of hyperbranched polymer-coated silica particles is the surface-initiated self-condensing vinyl polymerization (SCVP) of a vinyl AB* inimer from an initiator functionalized silica surface. 2-(2-bromopropionyloxy)ethyl acrylate (BPEA) [66] and p-chloromethyl styrene (CMS) [56] (Table 3) have been employed as inimers to grow dendritic structures from the nanoparticle surface. These core-shell particles were used for the polymerization of a second monomer, initiated by the end-groups at the outermost surface of the hyperbranched polymer shell, to obtain superstar structures bearing linear hydrophobic or polyelectrolyte outer chains which are attractive colloidal systems for use in surface coatings and paints.

**Table 3:** Inimer molecules employed for the synthesis of grafted hyperbranched polymers on silica particles by self-condensing vinyl polymerization.

No	Inimer	Ref.
1	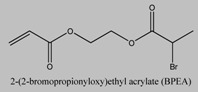	[66]
2	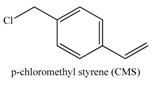	[56]

Lately, a new versatile method for the synthesis of industrially relevant branched polyolefin brushes, of a narrow molecular weight distribution, onto silica particles has been developed. The synthesis employed the surface functionalization of silica with acrylate moieties, which are used as anchoring sites for the covalent attachment of Pd-diimine complexes. The silica-supported Pd-diimine complexes catalyze the surface-initiated “living” polymerization of ethylene at 5 °C and an ethylene pressure of 400 psi to yield well-defined grafted polyethylene [96].

##### 2.1.2.3. Responsive nanohybrids

###### pH- and temperature-sensitive core-shell systems

Recent advances in the field of responsive materials focus on the coating of flat or curved surfaces with polymers exhibiting a stimuli-sensitive behavior and changing their properties in response to an applied stimulus. External stimuli, *i.e.*, temperature, pH and solvent quality, have been investigated leading to changes in the grafted polymer chain conformation and the physicochemical properties of the surface. Silica nanoparticles coated with responsive polymers are attractive building blocks for the development of “smart”, environmentally responsive nanostructured materials. The most extensively studied system is PNIPAM-grafted polymer chains onto silica nanoparticles which were shown to exhibit a non-conventional temperature-induced double phase transition behavior [60]. The phase transition at lower temperature is ascribed to the cluster-induced collapse of the inner region of the PNIPAM brushes, close to the silica surface, which posses a high chain density, while the transition at higher temperature is attributed to the outer region of the PNIPAM shell where the chain density is lower. The thermo-induced phase transition of the grafted polymer chains is shifted to lower temperatures and occurs over a broader temperature range compared to that of the free polymer in solution. This is attributed to interchain interactions in the brush layer [63]. The characteristic dehydration of temperature-sensitive polymers in densely-packed brushes can be crucial for the application of such systems as a thermo-responsive stationary phase in chromatography for the separation of active molecules. It was shown that the retention time of steroids increased over a broad range of temperatures for highly grafted PNIPAM-silica hybrids, while at lower grafting densities the retention time increased only in a narrow range around the LCST of the polymer [68]. 

The surface-initiated polymerization of responsive monomers has also been used for the modification of the pore properties of mesoporous materials. PNIPAM chains were grown in the pore walls of mesoporous silica leading to materials with temperature responsive pore properties. The long-range ordered hexagonal mesostructure and the thermo-responsive behavior of PNIPAM were retained after the synthesis of the interpenetrating silica-PNIPAM network rendering these materials attractive for use in bioseparations or molecular transport switches [97].

Another interesting effect induced by the stimuli responsive behavior of the polymer coating is the reversible transportation of stimuli-sensitive core-shell hybrids between two immiscible phases due to the reversible hydration-dehydration of the polymer shell. The reversible migration of temperature-sensitive polymer-grafted silica nanoparticles from an aqueous to an immiscible organic phase upon heating and cooling the system, respectively, was shown [64,98], which is consistent with the de Gennes concept [99].

Recently, it was proposed that pH-sensitive core-shell structures can be used as transducers of signals in logic operation systems. More specifically, “smart” signal-responsive hybrid systems with built-in Boolean logic were developed by grafting poly(2-vinyl pyridine) (P2VP) chains onto the surface of silica particles. The hybrids were coupled with an enzyme-based system and were used to detect signals induced by the enzyme function. The biochemical input signals, that is pH changes originated from the AND/OR logic operations of the enzyme systems, were detected by the self-assembly changes of the pH-sensitive hybrids in solution [100] rendering these materials very promising for use in the development of biochips and other biomedical devices.

Another responsive system of particular interest are “hairy” silica nanoparticles comprising mixed homopolymer brushes. These hybrids possess interesting responsive properties induced by changes of the solvent quality. To ensure the uniformity of the mixed polymer layer on the surface of the nanoparticles Y-shaped multifunctional colloidal initiators carrying dual initiating functionalities have been employed. A combination of ATRP followed by NMP chemistry resulted in the formation of the mixed brush by sequential polymerization. The phase behavior of the mixed poly(*t*-buyl acrylate) (P*t*BA)/PS brushes on the silica nanoparticles showed a lateral phase separation of the two polymers under equilibrium melt conditions which resulted in worm-like patterns with a feature size of around 10 nm [62]. Furthermore, hydrolysis of the *tert*-butyl groups produced amphiphilic mixed poly(acrylic acid) (PAA)/PS brushes which rendered the particles dispersible in both chloroform which is a selective solvent for the PS chains and in methanol which is selective for the PAA grafts *via* the chain reorganization on the surface of the “hairy” particles [61].

###### Photo-responsive hybrids

Core-shell hybrids carrying photo-responsive groups, whose properties can be reversibly altered by applying a remote stimulus such as light of specific wavelength, are highly attractive nowadays. Poly(spirobenzopyran)-*co*-poly(methyl methacrylate) (PSP-*co*-PMMA) copolymer brushes have been synthesized onto the surface of silica particles by ATRP. A progressive decrease of both the coloration and decoloration rates of the SP moieties incorporated in the polymer chains was observed, accompanied by the H-stacking of the chromophore moieties in dense polymer brushes upon irradiation [65]. This effect is very attractive and has been used for the formation of two-dimensional and three-dimensional structures by a light induced assembly and phase separation of the hybrid nanocolloids. The latter is based on the UV induced isomerization of the spiropyran (SP) moieties to merocyanine (MC) groups which exhibit a high dipolar moment and form SP-MC interchain complexes in less polar media leading to the aggregation of the particles. Both the SP content of the polymer and the solvent polarity were shown to affect the photo-induced aggregation of the polymer shell [101]. Two dimensional-patterned colloid assemblies were obtained *via* the appropriate modification of a flat substrate with a PSP-*co*-PMMA polymer film which was next immersed in a stable colloidal dispersion of the photo-responsive hybrids. Upon UV irradiation, the MC moieties formed on the flat surface due to the SP photo-isomerization, aggregate with the SP moieties of the polymer brushes of the colloidal hybrids and hold the two surfaces together [102]. Analogous three-dimensional porous structures have been fabricated by direct laser writing of a dense colloidal suspension of the photo-responsive spheres. A two photon absorption process at a focal point of a near-IR pulsed laser, focused tightly in the colloidal dispersion, induce gelation of the hybrids and the isomerization of the SP photo-responsive moieties of the polymer brush to MC groups. The MC-SP and MC-MC *H*-aggregates formed, induce strong intermolecular and interparticle bonding in the system and lead to the 3D structure fabrication [103]. Recently, a similar photo-responsive core-shell hybrid system was proposed for use as a sensitive ratiometric fluorescent thermometer. The hybrid comprised silica particles coated with a densely grafted PNIPAM shell, labeled with a fluorescence resonance energy transfer (FRET) donor in its inner layer and a photo-responsive SP acceptor in the outer layer. Upon irradiation with UV the non-fluorescent SP form isomerizes to the fluorescent MC form and facilitates the FRET process between the donor and the acceptor moieties. The thermo-modulated collapse/swelling of the PNIPAM shell can be used as a temperature probe which tunes the relative distance of the donor and the acceptor in the polymer shell and influences the FRET efficiency [59]. 

Finally, photolabile polymer shells have been used to tune the surface wettability of nanocolloids upon irradiation with light. Photolabile hydrophobic coatings were efficiently transformed to hydrophilic shells thus switching the surface from hydrophobic to hydrophilic. The switch can be augment by increasing the surface roughness leading to tunable superhydrophilic/superhydrophobic surfaces [104]. 

###### Hybrids with gating properties

An ongoing challenge in materials science relies on the development of hybrid assemblies displaying gating properties. It was recently shown, that transport through colloidal nanoporous assemblies can be effectively modulated by modifying the surface of the colloids with “smart”, environmentally-responsive polymer brushes in such a way to mimic the gating function in biological nanoporous systems. The first example in this field was reported by Schepelina and Zharov. They used poly(acrylamide) brushes grown by ATRP to modify the surface of the nanopores in films of closed-packed face-centered cubic (fcc) lattices of submicrometer silica spheres. Flux measurements of redox species across these opal films verified that the polymer film thickness increased with the polymerization time leading to a decrease of the pore size [70]. The use of temperature-responsive PNIPAM brushes allowed tuning the flow as a function of temperature, while the film thickness had also a significant effect on the diffusion through the nanopores. Nanopores bearing a thin PNIPAM layer possessed a positive gating behavior and the diffusion rates increased with increasing temperature, while the nanopores with the thicker PNIPAM layers exhibited the opposite effect (negative gating behavior) and diminished the flow through the nanopores at higher temperatures due to the dehydration of the densely packed polymer inside the nanopores [71]. An alternative approach to develop “smart” hybrid materials displaying gating properties involves the simple modification of ordered mesoporous networks or porous nanocolloids with stimuli responsive end-grafted polymer chains. The temperature-dependent uptake and release of small molecules by these porous materials was modulated by the polymer phase transition and the porosity of the materials. The presence of the hydrated and extended polymer within the porous structure at low temperature inhibited the transport of solutes, while at higher temperatures the collapse of the hydrophobic polymer in the globule conformation within the pores allowed solute diffusion [105]. The inherent disadvantage of the above system was the leakage of the active molecules even at low temperatures. To overcome this problem a different mode of action has been targeted which relies on the uptake and release of the actives below rather than above the LCST. This was achieved by “grafting to” temperature-responsive polymer chains onto the surface of micro-to-mesoporous silica nanoparticles. Below the LCST, the grafted polymer chains adopted a random coil configuration and facilitated the uptake and release of the molecules through the pores. However, upon increasing the temperature above the LCST the polymer chains collapsed, blocked the pores and retarded the diffusion of the molecules [106].

Finally, pH-responsive polymer coatings exhibit a rather complex pH- and ion-responsive flux [107] behavior which is dependent on the pH, the charge of the active species and the ionic strength of the medium have been developed as gating materials. The stretching of the polymer chains due to the ionization of the monomer repeat units block the nanopores and decrease the diffusion rate. The flow can be restored by increasing the salt concentration which screens the electrostatic repulsion forces [57]. pH-responsive polymer shells were proposed for the development of organic-inorganic hybrid membranes exhibiting high proton conductivity. To this direction, sintered silica and self-assembled nanoporous silica colloidal crystals modified with surface-bound polymer brushes carrying acidic groups were shown to exhibit high water uptake and proton conductivity. However, despite the superior proton conductivity of the self-assembled membranes their poor mechanical properties limit their use for practical purposes [58]. 

###### Pickering emulsions and Janus nanohybrids

Janus particles, which are characterized by an asymmetric nature in structure, have attracted particular attention, because they can self-assemble into fascinating hierarchical structures and serve as building blocks for complex superstructures. The preparation of such synthetic asymmetric particles is effectively assisted by Pickering emulsions, which are emulsions stabilized by particles rather than surfactant molecules. Pickering emulsions can be effectively stabilized by hybrid core-shell particles based on strong polyelectrolyte brushes grown from the surface of silica colloids. Anionic poly(styrenesulfonate) chains have been used because they are “quenched” [108] and highly charged and can thus form long-term stabilized oil-in-water emulsions which are stable against coalescence even at low particle concentrations. The emulsifying properties of the particles are based on the hydrophilic character of the polymer charged side-groups and the hydrophobic character of the polymer backbone which drive their absorption at the oil/water interface [69]. Amphiphilic Janus particles were prepared in a one-step synthesis by the adsorption of azide-modified silica spheres around the monomer droplets of a styrene/water emulsion with one hemisphere being immerse in the aqueous phase and the other in the organic phase. The emulsion-assisted synthesis of amphiphilic Janus particles comprising PS- and poly(sodium methacrylate)-coated hemispheres used initiator-modified particles to simultaneously grow the polymer chains from the surface of the particles in the two hemispheres. These hybrids can form supramolecular assemblies upon dispersion in solvents being selective for only one hemisphere [109]. In another approach, azide-modified silica particles have been used for the preparation of Janus particles using *in situ* click chemistry. Adsorption of these particles at a polymer/solvent interface allowed first the binding of one type of alkylated molecules on the solvent exposed hemisphere whereas next, the removal of the polymer template exposed the second hemisphere for the immobilization of a second alkylated polymer *via* click reaction with the residual azide groups. Using this general protocol both small molecules and long polymer chains can be introduced on the two particle hemispheres. Zhang *et al.* conjugated biotin molecules and poly(ethylene oxide) (PEO) chains onto azide functionalized silica particles. The aggregation of the biotin-conjugated hybrids upon the addition of avidin in solution, verified the bioavailability of the biotin-moieties to avidin which disposes four biotin-binding sites and can act as a cross-linker between the particles [53]. 

A challenge in the development of Janus particles is the ability to affect their self-organization properties by the application of an external stimulus. Following the Pickering emulsion approach of Granick and co-workers [110], stimuli-responsive oppositely charged Janus nanoparticles were synthesized using a sequential “grafting from”/“grafting to” approach to decorate the particles with temperature and/or pH responsive polymer chains, respectively. These switchable Janus particles form hierarchically structured aggregates upon altering the solution pH and are proposed for use in the controlled stabilization of emulsions and the regulation of molecular transport across the interface of two immiscible phases [54].

### 2.2. Polymer-grafted Silsequioxane nanoparticles

A lot of synthetic effort has focused on the preparation and properties of polyhedral oligomeric silsesquioxanes (POSS)-polymer hybrid materials due to the observed enhancement of the properties of the organic matrix (increased thermal stability, reduced flammability and dielectric constant) upon incorporation of POSS molecules [111,112,113,114,115,116]. POSS molecules have a cage-shaped three-dimensional structure with a general chemical formula (RSiO_1.5_)*_n_*. Among them, octasilsesquioxanes (R_8_Si_8_O_12_) (*n=*8) have been widely investigated owing to their precise molecular structure; they consist of a rigid, cubic silica core with a 0.53 nm side length, and an organic group attached to each of the eight corners. These organic groups can be either inert or reactive, and control the reactivity and the solubility of the POSS molecules [117,118]. Intense research effort has recently led to the preparation of POSS-containing hybrid polymers of different topological structures such as star-shaped, block and tadpole-shaped hybrid polymers. Telechelic and hemitelechelic POSS-containing hybrids have been also synthesized.

#### 2.2.1. Star-shaped hybrids

Star-like hybrids with inorganic POSS cores can be prepared by the “core-first” method in which the polymerization is initiated by appropriately functionalized POSS molecules. Well-defined end-tethered homopolymers and block copolymers were grown from the polysilsesquioxane (SiO_1.5_) nanoparticles by ATRP leading to layered core-shell structures [119,120]. A similar method employed POSS macroinitiators with about 58 functions to grow water soluble glycopolymer functionalized silsequioxane particles of approximately 25 arms. Such well-defined branched structures are attractive for a wide range of applications in biosciences as well as hybrid thickeners in coatings and paints.

An interesting approach was followed for the synthesis of a unique hybrid architecture comprising a POSS core surrounded by four macrocyclic polymer arms to form quatrefoil-shaped star-cyclic PS. The synthesis involved a combination of ATRP chemistry to form an 8-arm star with a POSS core and eight end-functionalized polymer arms carrying azide functionalities. Click reaction between the azide functionalities and a difunctional linker yielded a polymer loop with both ends attached on the POSS molecule. The star-cyclic PS processed a higher glass transition temperature compared to the star-linear PS due to the absence of chain ends in the former molecule [121]. These molecules are also expected to exhibit interesting rheological properties and dynamics related to the ring polymer structure.

#### 2.2.2. Tadpole-shaped hybrids

An interesting POSS-based hybrid architecture combines a particle with one covalently bound polymer chain in a single entity. Initial work focused on the growth of methacrylate polymers from a functionalized POSS however, low degrees of polymerization [122] were obtained attributed to the steric hindrance by the bulky POSS molecule [123]. Recently, tadpole-shaped polymeric hybrids with an inorganic POSS “head” and a well-defined polymer “tail” were synthesized using an incompletely condensed POSS molecule bearing a highly reactive trisodium silanolate group as the efficient and versatile initiator for copper-mediated ATRP. This allowed the synthesis of POSS-polymer hybrids with good control over the polymer molecular weight and molecular weight distribution and gave hybrid materials with enhanced degradation and glass transition temperature compared to those of the model polymers without the POSS moiety [124]. POSS-PMMA block copolymer hybrids have been employed for the preparation of low-dielectric-constant, nanoporous thin films by the sequential secondary condensation of the silanol groups and the decomposition of PMMA which led to pore formation within the film [125]. It was also shown that PMMA-POSS hybrids exhibit specific interactions with phenolic resins to produce totally miscible amorphous blends in which at least three competing association mechanisms take place: self-association of the phenolic groups (hydroxyl-hydroxyl interactions), hydroxyl-siloxane inter-association between the phenolic moieties and POSS, and hydroxyl-carbonyl inter-association between the phenolic groups and PMMA [126]. POSS-diblock copolymer hybrids can form well-defined self-assembled long-range lamellae structures [127]. The formation of these hierarchical nanostructures is driven by the low polydispersity and the high molecular weight of the polymers synthesized by living anionic polymerization. The morphology of POSS-based block-copolymer thin films showed vertically oriented lamellae and cylinders [128] which could be tuned to spheres, cylinders, and lamellae by varying the grafted polymer chain length. A helix-like structure was also formed to relieve the steric crowding of the POSS units which upon thermal annealing packed into an orthorhombic lattice structure driven by POSS aggregation [129]. 

Tadpole-shaped fluorinated POSS molecules bearing well-defined grafted PMMA chains were blended in a PMMA matrix and were shown to migrate at the air/polymer interface with the outermost layer of the film covered almost completely by the POSS heads. Owing to this unique structure, the films exhibited strong resistance against Ar+ ion etching, despite their low overall POSS content [130] and are attractive for various applications. 

Recently, responsive polymer-POSS hybrids comprising temperature-responsive or polyelectrolyte polymer chains have been prepared leading to amphiphilic tadpole-shaped structures. These molecules self-assemble into well-defined temperature-responsive POSS-core micelles or pH-responsive particulate aggregates in aqueous media [131,132] and are proposed for use in controlled drug delivery. 

#### 2.2.3. Telechelic and hemitelechelic POSS-containing hybrids

Telechelic polymers are linear chains containing associating ‘sticker’ groups only at the chain-ends and are analogous to triblock copolymers which self assemble in selective media. Telechelic and hemitelechelic POSS-containing hybrid polymers have been prepared by direct urethane linkage between the hydroxyl end-groups of the polymer and the monoisocyanate groups of the POSS molecules [133]. The use of a hydrophilic polymer resulted in the formation of amphiphilic telechelics the hydrophilic/hydrophobic balance of which was controlled by the molecular weight of the polymer. In bulk, the bulky nature of POSS influences the crystallization of the polymer with respect to the crystalline lamellae dimensions [134] while POSS moieties, attached to both polymer ends aggregate and form nanocrystals [135]. In dilute solution the POSS-polymer telechelics associate to form nanostructures which are influenced by the polymer architecture and the solvent polarity [136].

### 2.3. Polymer-grafted metal-oxide semiconducting nanoparticles

The functionalization of low-dimensional semiconductor materials such as metal oxide (TiO_2,_ ZnO, SnO_2_) nanoparticles, nanotubes and nanowires with grafted polymer chains has attracted particular attention owing to their intriguing physical properties and their potential use in a wide range of applications. For example hybrid TiO_2_ particles can be incorporated into capacitors and thin film transistors leading to the enhancement of the dielectric constant (K) and to mobilities approaching 0.2 cm^2^/V×s [137]. However, the surface chemistry of these materials is rather complex and thus the immobilization of small organic molecules or long polymer chains, is not necessarily straightforward.

#### 2.3.1. Polymer-grafted TiO_2_ nanoparticles

The surface of TiO_2_ nanoparticles can be modified by the “grafting through” the “grafting from” or the “grafting to” method. The latter requires pre-prepared polymer chains bearing an appropriate anchoring group. Dopamine moieties have been the most efficient anchoring groups used so far allowing the attachment of long stabilizing polymer chains which lead to the effective dispersion of the TiO_2_ nanoparticles in organic media [138,139,140].

**Scheme 1:**
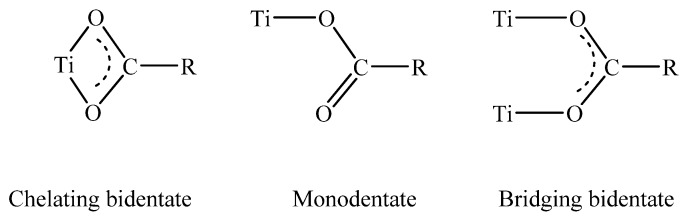
Coordinating modes of the carboxylate groups on the titania surface.

The use of initiator functionalized particles has been also investigated for the growth of polymer coatings directly from the nanoparticles surface (Table 4). However, the surface chemistry of the TiO_2_ particles is different to that of silica and thus the development of new initiator anchoring groups is required. The attachment of organic moieties to the surface of these particles by strong covalent bonding requires the use of highly reactive organic groups such as carboxylic acid [141,142,143,144], phosphoric acid [145,146,147] and catechol derivatives [148]. Recently, an approach involving the reaction of an acid chloride-terminated initiator with the nanoparticle surface was also applied for the immobilization of an ATRP initiator onto the nanoparticles surface [149]. Three possible coordination mechanisms have been proposed for the binding of the carboxylic acid groups onto the TiO_2_ surface; the monodentate, the chelating and the bridging coordination mode (Scheme 1). The preferred coordination mechanism is highly dependent on the nanoparticle size which affects the coordinate states of the surface Ti atoms of the nanoparticle. Biomimetic catechol binding sites are advantageous because they facilitate the intramolecular ligand-to-particle charge transfer within the surface Ti-catechol complex [150]. This approach has been used for the growth of metal-binding polymer chains on the surface of the titania nanoparticles followed by the templated growth of metal nanoparticles within the polymer shell to prepare a ternary nanostructure with electrocatalytic properties [151].

Another route for the preparation of initiator-functionalized metal oxide nanoparticles involves the use of metal alkoxides appropriately derivatized to carry polymerization initiating sites. Upon hydrolysis and condensation of these precursor molecules, amorphous titanium, zirconium and vanadium oxo clusters bearing surface initiating sites are obtained which can serve as multifunctional initiators for si-ATRP [152,153].

#### 2.3.2. Polymer-grafted ZnO nanoparticles

Si-ATRP has been employed for the functionalization of ZnO nanoparticles with grafted polymer chains. The nanoparticles were pre-functionalized with an appropriate organic initiator using two different anchoring chemistries (Table 4); silane condensation or carboxylic acid coordination. 

Both hydrophobic [156] and hydrophilic [155,157] polymers have been grown from the surface of the ZnO nanoparticles by ATRP. Moreover, hyperbranched polymer-functionalized ZnO particles were obtained by surface-initiated self-condensing vinyl polymerization (si-SCVP) from initiator-functionalized nanoparticles [154]. 

However, the surface functionalization of metal-oxide nanoparticles is far from being resolved. There are undoubtedly still many challenges to be meet in the synthesis, functionalization, characterization and potential applications of these materials.

**Table 4:** Initiator molecules grafted on the surface of TiO_2_ and ZnO nanoparticles.

No	Initiators	Polymer	Ref.
1	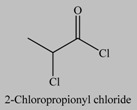	POEMA, PSS	[149]
2	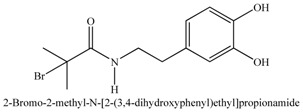	PMMA, PDMAEMA	[150,151]
3	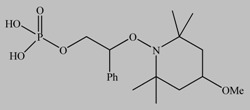	PS, P3VP	[146,147]
4	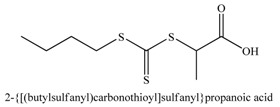	PAA	[142]
5	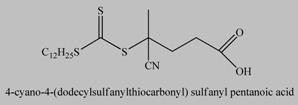	PMMA	[143]
6*	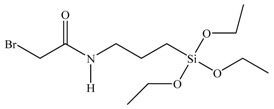	PHEA	[154,155]
7*	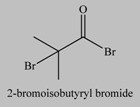	MeO-PEGMA, PMMA, PCEMA	[156,157]

* Initiators used for ZnO functionalization.

### 2.4. Polymer-grafted magnetic nanoparticles

Magnetic nano-objects have been deemed to hold immense promise for use in *in vivo* biomedical applications including magnetic resonance imaging (MRI) contrast enhancement [158,159,160,161], targeted drug delivery [162,163], hyperthermia [164,165], magnetic field assisted radionuclide and cancer therapy [166], *in vitro* automated separations and isolation of biomolecules and cells [167,168,169] and magnetically separable nanocatalytic systems [170,171]. Recently, magnetic nanoparticles have been used to selectively separate “living” from dead polymer chains prepared by a RAFT process using Z-functional RAFT agents with high affinity for the surface of the nanoparticles [172]. However, due to van der Waals attraction forces and their anisotropic dipolar attraction, the magnetic nanoparticles tend to agglomerate into larger clusters and the properties associated with their nanometer dimensions are eliminated. Their functionalization with organic stabilizers [173,174] can prevent aggregation and improve their dispersion and the confinement of the inorganic material in a polymer matrix [175,176]. 

Several reports have described the surface functionalization of magnetic nanoparticles with initiating sites (Table 5) for the growth of polymeric stabilizers utilizing CRP techniques and showed that the magnetic properties were not significantly affected [177,178].

Lattuada and Hatton proposed a flexible methodology for the preparation of magnetic nanoparticles coated with poly(lactic acid) end-tethered chains grown by surface-initiated ROP and poly(methacrylate) end-grafted chains obtained by ATRP [179]. Poly(2-dimethylamino)ethyl methacrylate) (PDMAEMA) end-grafted chains were also synthesized from the surface of γ-F_2_O_3_ nanoparticles by ATRP affording an “amphibious” character to the hybrids. These core-shell nano-objects being soluble in both aqueous and common organic solvents remain in the bulk phase in water/oil biphasic systems [180]. PS- and PAA-functionalized F_3_O_4_ nanoparticles were also obtained by a RAFT-mediated process utilizing peroxide and hydroperoxide functionalities generated on the surface of the nanoparticles upon ozone pretreatment [181]. F_2_O_3_ [182] and MnFe_2_O_4_ [183] core-PS shell hybrids were synthesized by surface-initiated ATRP utilizing an initiator bearing a carboxyl anchoring group. However, the non-covalent linkage of the macromolecules onto the surface facilitated the exchange of the polymer chains with other competing molecules from the solution possessing –COOH groups. One way to overcome this problem is by cross-linking the PS chains onto the nanoparticle surface [184]. 

**Table 5:** ATRP initiators anchored on the surface of magnetic nanoparticles.

No	Initiators	Ref.
1	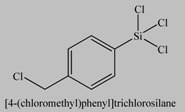	[185]
2	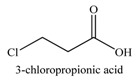	[183]
3	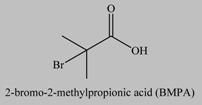	[180,182,186,187,188,189]
4	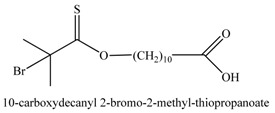	[184]
5	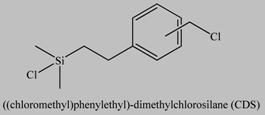	[177]

Recently, Xu *et al.* studied the self-assembly process of nanocomposite films consisting of a lamellar-forming PS-*b*-PMMA copolymer and PMMA-coated magnetite nanoparticles as a function of the concentration of the nanoparticles and the molecular weight of the polymer chains [190]. The particles coated with short PMMA chains were dispersed at low particle concentration and aggregated only upon increasing the concentration of the nanoparticles. However, the increase of the molecular weight of the end-tethered PMMA chains induced nanoparticle aggregation even at low particle concentration. The size of these aggregates was larger than the copolymer domain size and forced the block copolymer to form an onion-like morphology around the aggregates. PMMA-coated magnetic nanoparticles synthesized by ATRP were also introduced in a thin polymer film of a symmetric P2VP-*b*-PMMA copolymer. The structural polymorphism of the film was studied in the presence of carbon tetrachloride vapors, leading to the formation of hexagonal and lamellar microphase-separated morphologies at different exposure times due to the selective solvation of the PMMA block in the solvent. The colloidal hybrids segregated in the PMMA domains of the diblock copolymer due to the affinity of the PMMA-shell for the PMMA block. However, the self-assembled morphology of the film disappeared upon application of a magnetic field generated by the interaction of a magnetized atomic force microscopy tip with the magnetic nanoparticles [191].

Tuning the composition and functionality of the macromolecules introduced onto the surface of the nanocolloids allows the tailoring of the novel physical, chemical and biological functions of the nanoparticles. In particular, the functionalization of Fe_3_O_4_ particles with poly(ethylene glycol methacrylate) (PEGMA) chains does not only improve the dispersability of the nanoparticles in an aqueous solution but also increases the in-vivo circulation time of the magnetic nanoparticles. PEGMA renders the surface neutral and hydrophilic and increases its resistance to the adsorption of proteins or macromolecules bearing receptors recognized by the macrophages, thus preventing the endocytosis/phagocytosis of the particles by the macrophase cells [185]. Fe_3_O_4_/SiO_2_-g-poly(styrene sulfonate sodium salt) hybrids were also proposed for use as solid supports for the immobilization of enzymes such as pectinase. Biochemical studies proved that the enzyme retained its activity for 30 days, at a wider pH and temperature range compared to the free enzyme [192].

A challenging issue that has emerged lately is the formation of clusters, comprising polymer-coated magnetic nanoparticles, in response to an external trigger. These clusters are advantageous for certain potential applications as for example in high gradient magnetic separation processes in which clustering enables the facile scavenging of the particles after the adsorption process [193]. The development of such “smart” hybrids which undergo relatively large and abrupt changes in their physical and/or chemical and colloidal properties in response to external environmental conditions requires the functionalization of magnetic nano-objects with a polymer shell being sensitive to an external stimulus such as the temperature, pH, ionic strength, magnetic field, *etc.* The thermo-responsive dispersion of F_3_O_4_@PHEMA hybrids in methanol [186], F_3_O_4_@PS particles in cyclohexane [188,189] and poly(*ε*-caprolactone) (PCL)-coated colloids in dimethyl sulfoxide [187] has been studied. The solubility of the polymer as a function of the solvent quality is determined by the enthalpy and the entropy of the system and is described by the Florry and Huggins theory [194]. Below a critical temperature known as the theta temperature (*T_θ_*) the polymer chains are in a collapsed state due to the predominant attractive forces between the polymer segments. At *T_θ_* the entropy determines the polymer chain conformation which behaves as an ideal chain. Above *T_θ_* the dominant forces are the repulsive interchain and the favorable chain-solvent interactions causing the polymer chains to expand. This thermo-responsive behavior of PS-coated superparamagnetic nanoparticles allowed the development of magneto-responsive Pichering emulsions in a cyclohexane/water mixture above the *T_θ_* of PS [188], which offer new opportunities for the encapsulation and controlled release of lipophilic compounds using the magnetic heating of the emulsion in an ac magnetic field to break the emulsion on demand. 

Of particular interest is the ability to control the aggregation of core-shell systems employing the property of several polymers to collapse in aqueous media upon heating above the LCST. This approach has been used to enhance the contrast in magnetic resonance imaging by introducing temperature sensitive Fe_3_O_4_ nanoparticles into blood cells and causing their reversible agglomeration in response to temperature changes [195]. The hybrid-loaded cells exhibited better magnetic response and enhanced magnetic contrast above the LCST due to aggregation. Besides the direct temperature change of the hybrid dispersion, the application of an AC magnetic field to generate heat, which is transferred to the temperature-responsive polymer-shell, can be also used to indirectly cause the aggregation of the nanocolloids [196]. A great challenge in this field is the ability to control the cluster size upon aggregation. Janus particles possessing stimuli responsive polymer coatings were shown to meet these requirements. Due to their asymmetric surface functionalization these nano-objects become amphiphilic and self-assemble into well-defined clusters in response to an external stimulus. Janus magnetite nanoparticles coated at one hemisphere with a pH-responsive polymer and on the second hemisphere with a pH-independent or a temperature-responsive polymer can self-assemble into micelle-like structures of finite-size at low pH or high temperatures driven by the individual response of each of the polymer coatings [197]. Moreover, polyampholyte Janus nanoparticles comprising acidic and basic hemispheres have shown an interesting pH-responsive behavior forming clusters at low pH-values due to the excess charge on the one hemisphere of the nanoparticles followed by aggregation and precipitation of the nanocolloids at intermediate pH-values due to charge neutralization on the particle surface [193].

### 2.5. Polymer-grafted metal nanoparticles

#### 2.5.1. Hybrid gold nanocolloids

Functionalized metal nanoparticles such as Au, Ag, Pt, and Cu have been the subject of intense research activity over the last decade. The unique electrical properties of these particles [198,199,200,201,202] as well as their optical and photophysical features (size-controlled plasmon absorbance, photonic electron–hole pair generation, and fluorescence) have attracted great attention [203,204]. Gold nanoparticles are probably the most attractive metal colloids for potential applications, in electronic and optical materials [205,206,207,208,209,210,211] and in biomedicine due to their biocompatibility and non-toxicity [212,213]. However, gold nanoparticles have a high tendency for aggregation which is usually accompanied by a red-shift and a broadening of their plasmon band thus limiting their use in the above applications [214]. The modification of the surface properties of gold nanoparticles was thus urged by the need to stabilize them in both organic and/or aqueous media. Polymeric stabilizers have been employed using either the “grafting to” or the “grafting from” approach and were shown to effectively prevent nanoparticle aggregation. Although the “grafting to” method is more facile and allows better control of the molecular weight and molecular weight distribution of the grafted polymer chains, the “grafting from” technique has lately emerged as the preferred method to prepare densely coated nanoparticles with enhanced colloidal stability.

##### 2.5.1.1. “Grafting to” approach

The “grafting to” approach almost exclusively employs the relatively strong sulfur-gold interactions to bind sulfur-terminated surfactant or polymers onto the gold surface. This method is particularly useful because it allows control over the molecular characteristics of the polymer ligand which is usually synthesized by a controlled/living polymerization technique prior to its grafting onto the nanoparticle surface. Among others, RAFT has emerged as a very promising controlled radical polymerization technique for the preparation of gold nanoparticle stabilizers not only because of its versatility and simplicity, but also because polymers prepared by the RAFT technique bear dithioester end-groups which are easily reduced to thiol end-groups in the presence of a reducing agent. Thiol-modified gold colloids are very stable and behave as robust large molecules being amenable to characterization by a variety of analytical methods.

There are several reports in the literature on the modification of gold nanoparticles with neutral, anionic, cationic and switterionic pre-synthesized polymers [215,216]. Wuelfing at al. first reported the grafting of thiol terminated PEO onto the surface of gold nanoparticles [217]. These nanohybrids can be useful as computed tomography (CT) contrast agents in blood pool and hepatoma imaging. Combination of the antibiofouling properties of PEG with the high X-ray absorption properties of the gold colloid provides an efficient CT contrast agent with a long circulation time that can avoid the shortcomings of current iodine-based CT contrast agents [218]. Thiol terminated PS synthesized by anionic polymerization have been also grafted onto the surface of gold nanoparticles and their homogeneous dispersion in a PS matrix was confirmed [219]. The use of faceted gold nanoparticles for the grafting of thiol-terminated hydrophobic or hydrophilic homopolymers allowed the grafting of very dense polymer brushes with grafting densities 1.2− to 23.5−fold greater than those accessible in a self-assembled monolayer onto two-dimensional gold surfaces [220]. An interesting approach for the synthesis of hydrophobic and hydrophilic arms uniformly attached in an alternating fashion on the surface of gold nanoparticles used a V-shaped polybutadiene-poly(ethylene glycol) (PB-PEG) amphiphile containing a carboxyl group at its junction point to graft the molecules onto mercaptophenol-functionalized gold nanoparticles *via* an esterification reaction. These V-shaped amphiphilc arms render the hybrid gold particles soluble in water as well as in organic media, and provide an exceptional thermal and solvent stability to the Au clusters, which remain in solution without precipitation, agglomeration, or decomposition for more than two years [221].

Temperature sensitive polymeric stabilizers have been extensively used for the modification of the surface of gold nanoparticles. PNIPAM homopolymers prepared by the RAFT technique using *S*-benzyl dithiobenzoate as the RAFT agent were immobilized onto gold particles by the “grafting to” method following reduction of the dithioester end-group with sodium borohydride to provide the required thiol group. These thermo-sensitive hybrids retained the LCST of PNIPAM and exhibited a sharp, reversible phase transition in solution between 25 and 30 °C [222]. The addition of salt was found to promote interparticle associations leading to a red shift in the plasmon band and an increase in the hydrodynamic size, attributed to aggregate formation [223]. This feature can be useful for the development of sensing devices due to the enhancement of the optical response obtained from the functionalized gold nanoparticles upon their interaction with specific substrates [224]. Responsive three-layer core-shell-corona microstructures are accessible by this method by grafting thiol-terminated responsive diblock copolymers onto the surface of gold nanoparticles [225]. These nanostructures can be shell cross-linked at high particle concentrations, due to the protection afforded by the hydrophilic corona, resulting in the formation of locked layered nanoparticles with improved colloidal stability. Particles exhibiting a complex pH- and temperature-responsive behavior and reversible swelling properties both before and after the cross-linking of the shell were obtained.

A facile approach to stabilize gold nanoparticles within a polymeric matrix employs the use of star block copolymers [226] or diblock copolymer micellar structures into which the nanoparticles are incorporated by simple polymer physisorption on the nanoparticle surface or hydrophobic interactions between hydrophobized particles and the micelle core [227]. Even though these core/shell structures can be permanently fixed by cross-linking the polymer shell a disadvantage of this method is found in the difficulty to control the shell thickness and the number of nanoparticles within each polymer nanostructure. 

##### 2.5.1.2. “Grafting from” method

The “grafting to” method described above often requires a large concentration of thiol-terminated stabilizers in solution which can interfere with the surface functionalization. Moreover, polymer adsorption onto nanometer-sized metal particles cannot usually provide compact core-shell systems because of steric hindrance among the adsorbed and the approaching polymer chains. Gold nanoparticle core nanostructures with a dense polymer layer of well-defined homopolymers and copolymers are easily prepared using a controlled/living polymerization technique to grow the polymer chains from initiator-functionalized gold nanoparticles (Table 6). 

**Table 6:** ATRP Initiators immobilized on the surface of gold nanoparticles.

No	Initiator	Polymer	Ref.
1	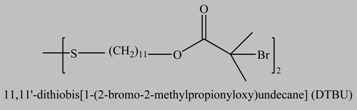	P4VP, PMMA, P(MeO-PEGMA)-*b*-PNIPAM, PNIPAM, PDMAEMA-*co*-PDEAEMA-*co*-P(PEGMA)	[228,229,230,231,232,233,234,236]
2	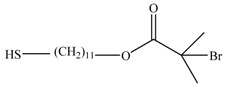	PMMA, P*n*BA, P*t*BA	[237,238,239]
3	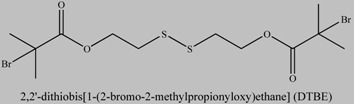	PDMAEMA	[234]

Watson *et al.* reported the transition-metal catalyzed ring-opening metathesis polymerization of norbornenyl-containing monomers carrying pendent electrochemical sensitive ferrocene groups from the surface of gold nanoparticles [240]. Dense polymer brushes were synthesized from the surface of gold particles by surface-initiated living cationic polymerization of 2-oxazolines in “one-pot multistep” reaction. For this, end-functionalized three-dimensional self-assembled monolayers (SAMs) on gold nanoparticles were used to initiate the living cationic ring-opening polymerization reaction directly on the gold nanoparticle surface. The resulting gold/polymer nanocomposite consisted of “brush-type” shells of linear macromolecules which upon the introduction of a terminal mesogen by means of a quantitative termination reaction resulted in an amphiphilic core-shell structures of well-defined hydrophilic/lipophilic balance [241]. Among the syntheses used, si-ATRP from self-assembled monolayers of bromofunctionalized thiolates on gold nanoparticles appears as the most versatile and facile method for the development of structurally controlled hybrids with a gold core of several nanometers in diameter and a shell of variable thickness composed of well-defined, dense polymer brushes [237,238]. These highly grafted hybrids exhibit an intense surface plasmon absorption band which is blue shifted when increasing the grafted polymer chain length suggesting the improved stabilization of the gold colloids as the size of the polymer stabiliser increases [230]. Moreover, they can form a two-dimensional array with a high degree of structural order due to their long range interparticle interactions which is comparable to the full length of the grafted polymer chains [231]. 

Among the different polymer shells, stimuli-responsive polymers are particularly attractive because they allow the manipulation of the optical signal obtained from the gold nanoparticles by external triggers. Cationic, pH-responsive polymer brushes grown from gold nanoparticles *via* si-ATRP, were shown to exhibit a reversible shrinkage of the polymer chains which wrap around the gold particle surface. A careful investigation of the pH-response of the grafted polymer chains revealed a two-step transition ascribed to the initial polymer collapse which induced nanoparticle agglomeration in a second stage [229]. These hybrids can serve as pH-stimuli bimetallic catalysts because they can entrap transition metal ions by their efficient coordinating segments followed by *in situ* metal reduction [228]. Polymer-coated gold nanoparticles have been also used for the development of pH-sensitive, colloidally stable, hydrophilic polymer capsules which are attractive for the controlled encapsulation and release of drug molecules. The capsules were obtained by first cross-linking the polymer shells followed by the cleavage of the Au-S bonds to remove the gold cores [234]. Temperature-responsive polymers, PNIPAM and poly(*N*-cyclopropylacrylamide) (PCYPAAM), exhibiting an LCST behavior have been also grown from gold nanoparticles using the ATRP [216] or RAFT polymerization technique. The optical properties of these gold particles can be varied by temperature, based on the thermal-responsive behavior of the polymer monolayer. During the collapse of the PNIPAM grafts the surface plasmon not only decreases in intensity but also blue shifts due to the decrease of the hydrophilicity of the nanoparticle surface as it becomes covered by the collapsed polymer [242].

Besides, the growth of linear polymers the synthesis of cross-linked temperature responsive gels onto the surface of gold nanoparticles has been explored. Gold nanoparticles coated with a cross-linked PNIPAM shell have been proposed for use in nanoparticle trapping using the thermo-modulated “breathing process” [235] of the polymer shell [236].

Moreover, Janus gold-nanoparticles with two types of different polymer chains decorated on the opposite sides of the nanoparticles are accessible. A novel approach was employed which combined a “solid-state grafting-to” method for the immobilization of crystalline polymer chains on one hemisphere and a “grafting-from” technique for the growth of a second type of polymer from the “free” surface of the nanoparticles. The symmetric decoration of the Janus hybrids was confirmed by the selective *in situ* formation of platinum nanoparticles on one of the hemispheres [239]. Although this solid-state approach has a higher throughput compared with self-assembled monolayers it is limited to the grafting of crystalline polymers in one of the two hemispheres.

Finally, binary thermo-sensitive nanocomposites can be obtained by the growth of block copolymers comprising two thermo-responsive blocks on the surface of the gold nanoparticles. These core-shell nanostructures possess two transition temperatures corresponding to the thermally induced conformational transition of the inner and the outer polymer, respectively. Upon cross-linking the polymer shells the nanostructures can be used as temperature-sensitive catalytic systems with tunable catalytic activity of the incorporated metal nanoparticles as a function of the solution temperature [232].

#### 2.5.2. Polymer-coated platinum nanoparticles

Similar to gold nanoparticles, platinum nanoparticles have a high surface-to-volume ratio and consequently a large fraction of metal atoms are exposed at the surface and are accessible to reactant molecules. Few reports in the literature have described the synthesis of polymer chains from the surface of platinum nanoparticles using the “grafting from” method in combination with a CRP technique. Recently, Carrot *et al.* have grown poly(butyl methacrylate) (P*n*BMA) chains from initiator-derivatized platinum nanoparticles using ATRP. Small-angle neutron scattering studies in solution showed that the hybrid structure is closer to a star architecture rather than a dense polymer corona surrounding the inorganic core. The formation of 2D arrays (Langmuir film) of these polymer-grafted-platinum hybrids at the air-water interface was shown, in which the interparticle distance was controlled in the range of tens of nanometers to a few nanometers, by the polymer chain length and the surface pressure [243].

### 2.6. Polymer-grafted quantum dots

Nanometer-sized semiconductor nanoparticles or quantum dots such as CdSe, CdTe, CdS, and ZnS have been extensively studied and still remain an active research area because of their unique size- and surface-dependent nonlinear optical, physicochemical, and electronic properties [244]. Due to their size in the nanometer range, quantum dots show interesting physical behavior which is totally different to that found in bulk materials. This effect is known as the quantum confinement effect. 

Because of these unique electronic and optical properties, including their broad absorption spectra, narrow absorption profiles and their discrete energy bands, semiconductor nanoparticles are candidates for many applications in sensors, solar cells, biological imaging, diagnostics, data storage, light-emitting diodes, single-molecule transistors, and substrates for biological tags and detection devices [245,246,247,248,249,250,251,252,253]. The modification of the surface of the nanoparticles with polymers is highly advantageous since the polymer endows the quantum dots with new physical and chemical properties including solubility, processability, chemical and biological stability, biocompatibility, and further functionalization possibility. 

#### 2.6.1. “Grafting to” method

The grafting of polymer chains onto the surface of quantum dots requires the appropriate end-functionalization of the polymer chains and/or the pre-functionalization of the surface of the nanoparticles. Initial studies have shown the grafting of preformed thiol-terminated PCL of controlled molecular weight and narrow molecular weight distribution onto the surface of CdS nanoparticles. The hybrids were highly stable both in solution, in films and in polymer matrices [254].

**Figure 3:**
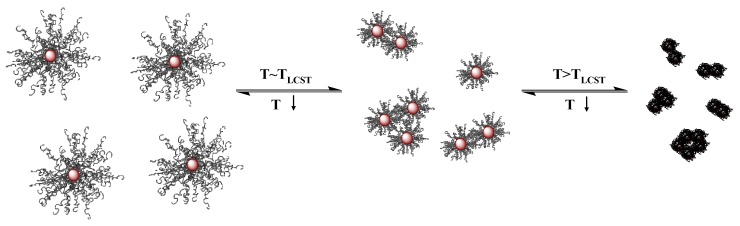
Reversible thermo-induced aggregation of temperature-sensitive hybrids.

Responsive PNIPAM-stabilized CdS particles have been prepared *via* the grafting of pre-synthesized thiolated-PNIPAM chains to the surface of CdS quantum dots. Association and dissociation studies of these hybrids induced by the increase and the decrease of the solution temperature, respectively (Figure 3), showed a hysteresis in the heating-cooling cycle attributed to the formation of large aggregates which initially dissociate into smaller aggregates as the temperature decreases, however, their complete dissociation is retarded by a balanced effect of inter- and intrachain hydrogen bonding as well as hydrophobic interactions between the PNIPAM chains and the CdS particles [255].

Recently, CdSe nanoparticles pre-functionalized with [(4-bromophenyl)methyl]dioctylphosphine oxide were used to covalently bind long vinyl-terminated poly(3-hexylthiophene) (P3HT) chains *via* a mild palladium-catalyzed Heck coupling. The solid-state emission spectra of the nanocomposites suggested an efficient charge transfer mechanism from the polymer to the quantum dots, which is three times faster compared to the energy transfer found for P3HT/CdSe composites prepared by physically mixing the pristine quantum dots in the polymer matrix [256]. 

Poly(*para*-methyl triphenylamine-*b*-cysteamine acrylamide) coated CdSe-ZnS quantum dots prepared by the “grafting to” method using thiol chemistry showed improved performance in light-emitting diodes attributed to the uniform film deposition of the hybrid layer as well as the facilitated hole injection into the quantum dots by the directly linked polymer [257]. 

#### 2.6.2. “Grafting from” approach

Conventional polymerization methods used to modify the surface of nanoparticles with polymers are not compatible with semiconductor nanoparticles. For example, anionic conditions are too severe for use in the presence of the quantum dots, and radicals generated by conventional radical polymerization (e.g., azo-bisisobutyronitrile initiated) degrade the nanoparticles and quench their photoluminescence. One of the first attempts for the surface modification of quantum dots came from Patten and Farmer who encapsulated CdS quantum dots in a silica shell and polymerized PMMA chains from the silica surface by ATRP [258]. These hybrid nanoparticles were casted into films which retained the photoluminescence of the precursor CdS nanoparticles and showed good dispersion of the polymer-coated CdS/SiO_2_ hybrids throughout the PMMA matrix. However, the latter advances in CRP techniques, which are accompanied with low radical concentrations, have allowed the growth of functional polymers from the surface of quantum dots.

“Unprotected” quantum dots without the intermediate silica layer have been used lately as substrates for the development of core-shell hybrids providing an intimate connection of the polymer to the nanoparticle core. Sill and Emrick reported the NMP synthesis of PS homopolymer and PS-*co*-PMMA copolymer chains from the surface of CdSe nanoparticles pre-functionalized with initiating molecules bearing phosphine oxide derivatives as the anchor groups [259]. The PS-coated CdSe nanoparticles not only showed a uniform dispersion in a polystyrene matrix but also exhibited enhanced fluorescence compared to the initiator functionalized particles due to their thermal treatment during the polymerization. At the same time a range of well-defined styrenes and (meth)acrylate homopolymers, random and block copolymers were grown by the RAFT technique from the surface of CdSe nanoparticles modified with trithiocarbonate moieties [260]. Upon surface modification with the polymeric stabilizers the particles maintained their optical properties without the requirement for a protective inorganic shell. 

Another approach used activators generated by electron transfer (AGET) ATRP in a miniemulsion system to synthesize well-defined polymer chains from the surface of pre-functionalized CdS nanoparticles [261]. The hybrids exhibited a high homogeneity, while quantum confinement effects were revealed by a blue shift at the onset of the trioctylphosphine oxide (TOPO) quantum dot absorption compared to the absorption of bulk CdS.

Polyolefin-coated CdSe nanoparticles have been prepared by ruthenium-catalyzed ROMP from particles pre-modified with *p-*vinylbenzyl-di-*n*-octylphosphine oxide [262]. This approach allowed the incorporation of nanoparticles of potential technological importance within simple and commercially available polymers and could potentially lead to industrially relevant applications. 

Moreover, an interesting approach was used to synthesize PCL *via* coordination-insertion ROP from the surface of CdS particles bearing hydroxyl-groups. In the first step, the reaction of mercaptoethanol and thioglycerol with cadmium acetate and thiourea afforded the hydroxy-coated CdS nanoparticles. The surface hydroxyl groups were next activated into aluminum alkoxide species, by reaction with AlEt_3_, which selectively initiated the ROP of (*ε*-caprolactone) from the nanoparticles surface. The covalent grafting of the hydrophobic polyester chains onto the semiconducting nanoparticles modified the surface properties of the nanoparticles and facilitated their dispersion in organic solvents such as toluene [11]. 

Hydroxyl-terminated germanium nanoclusters have been also appropriately functionalized to yield polymer-coated nanoclusters *via* ATRP. The original nanoclusters retain their photophysics upon altering the termination group which is consistent with a stable nanocluster surface [263]. 

Hyperbranched polyglycerol-modified quantum dots which are attractive for use in biomedical applications due to the reactivity of the hydroxyls towards functional biomolecules have been prepared by *in situ* anionic ROP from the surface of CdTe nanoparticles. Their good dispersion in water along with the strong fluorescence signal supported their use in cytotoxicity studies, which revealed the low toxicity of the hybrids in human lung cancer cells [264]. 

Finally, thermo-responsive quantum dots with PDMAEMA-grafted polymer chains, grown by surface-initiated oxyanionic vinyl polymerization, were shown to self-assemble into large spheres above the LCST of the grafted polymer and are attractive for use as “smart” materials in biological applications [265].

## 3. Conclusions and Perspectives

This review has given an overview of the range of hybrid core-shell nanostructures that can be prepared by end-grafting polymer chains onto the surface of inorganic nanocolloids using the direct covalent “grafting to”/“grafting from” strategies. CRP techniques have allowed the synthesis of well-defined advanced functional materials. It was shown that different mechanisms are possible and have been successfully applied for a variety of nanoparticles. However, although the surface functionalization of some types of nanoparticles such as silica and gold, have been extensively studied and, to a great extend, understood, more research is required for the successful dispersion and functionalization of other nanoparticulate objects, *i.e.*, semiconducting metal oxides, magnetic and quantum dots, which are still prone to aggregation and colloidal or chemical instabilities at various stages of their synthesis. Moreover, the grafting of inert polymer chains has been adequately described in most cases, whereas stimuli-responsive polymer brushes and their potential to control the designed morphology of the nanocomposite have not been fully explored. The controlled synthesis of end-grafted macromolecules with pre-defined molecular weight, composition, functionality and grafting density onto the surface of active nanocolloids will open up new avenues for their use in many high-value applications. Furthermore, much can be gained by understanding the structure-properties relationship of the nanohybrids for the retro-design of materials with the desired macroscopic properties.

## Abbreviations

AGET: activators generated by electron transfer
ATRP: atom-transfer radical polymerization
BPEA: 2-(2-bromopropionyloxy)ethyl acrylate
CMS: p-chloromethyl styrene
CRP: controlled radical polymerization
CT: computed tomography
FRET: fluorescence resonance energy transfer
LbL: layer-by-layer
LCST: lower critical solution temperature
MC: merocyanine
MeO-PEGMA: methoxy-poly(ethylene glycol) methacrylate
NMP: nitroxide-mediated radical polymerization
P(MeO-PEGMA)-*b*-PNIPAM: poly(methoxy-poly(ethylene glycol) methacrylate)-b-poly(*N*-isopropylacrylamide)
P2VP: poly(2-vinyl pyridine)
P3HT: poly(3-hexylthiophene)
P3VP: poly(3-vinyl pyridine)
P4VP: poly(4-vinyl pyridine)
PAA: poly(acrylic acid)
PAN: polyacrylonitrile
PB-PEG: polybutadiene-poly(ethylene glycol)
PCEMA: poly(2-(carbazol-9-yl)ethyl methacrylate)
PCYPAAM: poly(*N*-cyclopropylacrylamide)
PDEAEMA: poly(2-diethylamino)ethyl methacrylate)
PDMA: poly(*N*,*N*-dimethylacrylamide)
PDMAEMA: poly(2-dimethylamino)ethyl methacrylate)
PEO: poly(ethylene oxide)
PHEA: poly(hydroxyethyl acrylate)
PHEMA: poly(2-hydroxyethyl mehacrylate)
PMA: poly(methyl acrylate)
PMMA: poly(methyl methacrylate)
P*n*BA: poly(*n*-butyl acrylate)
P*n*BMA: poly(butyl methacrylate)
PNIPAM: poly(*N*-isopropylacrylamide)
POEMA: poly(oxyethylene methacrylate)
POSS: polyhedral oligomeric silsesquioxanes
PS: Polystyrene
PS-*b*-P*n*BA: polystyrene-*b*-poly(*n*-butyl acrylate)
PSP-*co*-PMMA: Poly(spirobenzopyran)-*co*-poly(methyl methacrylate)
PSS: poly(styrene sulfonic acid)
P*t*BA: poly(*t*-buyl acrylate)
RAFT: reversible addition-fragmentation chain transfer
ROMP: ring-opening metathesis polymerization
ROP: ring-opening polymerization
SAMs: self-assembled monolayers
SCVP: self-condensing vinyl polymerization
SFRP: stable free radical polymerization
si-ATRP: surface-initiated atom-transfer radical polymerization
SP: spiropyran
TOPO: trioctylphosphine oxide
UCST: upper critical solution temperature
